# Predicting Perioperative Venous Thromboembolism in Japanese Gynecological Patients

**DOI:** 10.1371/journal.pone.0089206

**Published:** 2014-02-26

**Authors:** Masae Ikeda, Hidetoshi Kan-no, Masaru Hayashi, Hitomi Tsukada, Masako Shida, Takeshi Hirasawa, Toshinari Muramatsu, Yoichi Ogushi, Mikio Mikami

**Affiliations:** 1 Department of Obstetrics and Gynecology, Division of Specialized Clinical Science, Tokai University School of Medicine, Isehara, Kanagawa, Japan; 2 Department of Medical Informatics, Division of Basic Medical Science and Molecular Medicine, Tokai University School of Medicine, Isehara, Kanagawa, Japan; Baylor College of Medicine, United States of America

## Abstract

**Objective:**

To develop a convenient screening method that can predict perioperative venous thromboembolism (VTE) and identify patients at risk of fatal perioperative pulmonary embolism (PE).

**Methods:**

Patients hospitalized for gynecological abdominal surgery (n = 183) underwent hematology tests and multidetector computed tomography (MDCT) to detect VTE. All statistical analyses were carried out using the SPSS software program (PASWV19.0J)

**Results:**

The following risk factors for VTE were identified by univariate analysis: plasmin-alpha2-plasmin inhibitor complex (PIC), thrombin-antithrombin III complex (TAT), and prolonged immobility (all p<0.001); age, neoadjuvant chemotherapy (NAC), malignancy, hypertension, past history of VTE, and hormone therapy (all p<0.01); and hemoglobin, transverse tumor diameter, ovarian disease, and menopause (all p<0.05). Multivariate analysis using these factors revealed that PIC, age, and transverse tumor diameter were significant independent determinants of the risk of VTE. We then calculated the incidence rate of perioperative VTE using PIC and transverse tumor diameter in patient groups stratified by age. In patients aged≦40 years, PIC ≧1.3 µg/mL and a transverse tumor diameter ≧10 cm identified the high-risk group for VTE with an accuracy of 93.6%. For patients in their 50 s, PIC ≧1.3 µg/mL identified a high risk of VTE with an accuracy of 78.2%. In patients aged ≧60 years, a transverse tumor diameter ≧15 cm (irrespective of PIC) or PIC ≧1.3 µg/mL identified the high-risk group with an accuracy of 82.4%.

**Conclusions:**

We propose new screening criteria for VTE risk that are based on PIC, transverse tumor diameter, and age. Our findings suggest the usefulness of these criteria for predicting the risk of perioperative VTE and for identifying patients with a high risk of fatal perioperative PE.

## Introduction

Venous thromboembolism (VTE) is a serious preventable complication of gynecological surgery and it has been known for decades that acute pulmonary embolism (PE) is relatively frequent in postoperative gynecological patients. VTE, comprising deep venous thrombosis (DVT) and PE, does not generally present with specific symptoms or findings and there are no simple definitive diagnostic procedures for these conditions. A recent study demonstrated the globally inadequate provision of VTE prophylaxis for acute inpatients [1]. Thus, there is a high incidence of VTE during hospitalization and it is still among the major postoperative complications and causes of death, even in the present era of supposedly advanced prophylaxis, diagnosis, and treatment.

Measurement of D-dimer is widely used at present. This test is highly sensitive and can accurately exclude VTE, but it has a low specificity and low positive predictive value, so patients with high D-dimer levels do not always have VTE [2]. Therefore, while measurement of D-dimer can be used to exclude VTE, it is necessary to find a new marker to reliably detect perioperative VTE.

Accordingly, the purpose of this study was to develop a convenient screening method that could predict perioperative VTE and identify patients with a high risk of fatal perioperative PE based on preoperative laboratory parameters, imaging findings, and patient background characteristics.

## Materials and Methods

The subjects were 183 ethnic Japanese patients who were admitted to Tokai University Hospital for planned gynecological abdominal surgery between October 2002 and March 2006. The patients underwent laboratory tests and multidetector computed tomography (MDCT) to detect VTE within two weeks before laparotomy, and MDCT was repeated prior to walking on the first day after surgery (Tables 1 and 2). We then followed the prognosis of these patients up to April 2012. Only patients having elective abdominal surgery were enrolled in this study, with those undergoing emergency surgery, laparoscopic procedures, and transvaginal operations being excluded.

**Table 1:** Characteristics of the 183 patients.

Characteristic	Mean ± SD
Age (years)	48.06±13.34
Body mass index (kg/m^2^)	23.21±3.87
Thrombin-antithrombin III complex (ng/mL)	6.53±8.73
Plasmin-alpha2-plasmin inhibitor complex (µg/mL)	1.22±1.56
Hemoglobin (g/dL)	12.15±1.77
Hematocrit (%)	37.43±5.05
Platelet count (×10^4^/µL)	27.11±8.67
Mean platelet volume (fl)	10.01±0.78
International normalized ratio	0.99±0.10
Longitudinal tumor diameter (cm)	11.35±5.80
Anteroposterior tumor diameter (cm)	9.23±4.41
Transverse tumor diameter (cm)	10.69±5.31
Operating time (minutes)	189.43±96.79
Blood loss (ml)	1000.79±1281.79

Abbreviation: SD, Standard Deviation.

**Table 2:** Characteristics of the 183 patients (continued).

Characteristic	yes	no	
Malignancy	78	105	
Transfusion	74	109	
Neoadjuvant chemotherapy	13	170	
Smoking	20	163	
Hypertension	26	157	
Diabetes	6	177	
Hyperlipidemia	12	171	
History of venous thromboembolism	1	182	
Varicose veins	1	182	
Menopause	67	116	
Hormone replacement therapy	1	182	
Prolonged immobility	2	181	
History of malignancy	4	179	
**Lesion**	**uterus**	**ovary**	**others**
	106	72	5

The Institutional Review Board for Clinical Research of Tokai University School of Medicine approved this study. Written informed consent was obtained at the time of enrollment and this study was performed in conformity with the Declaration of Helsinki.

To identify risk factors for VTE, the following variables were evaluated by univariate analysis (Mann-Whitney U test) to assess the relation with the risk of VTE occurring: plasmin-alpha2-plasmin inhibitor complex (PIC) (µg/mL), thrombin-antithrombin III complex (TAT) (ng/mL), age (years), body mass index (BMI) (kg/m^2^), hemoglobin (g/dL), hematocrit (%), platelet count (×10^4^/µL), mean platelet volume (MPV) (fl), international normalized ratio (INR), longitudinal/transverse/anteroposterior tumor diameters (cm), operating time (min), blood loss (g), malignancy (present/absent), site of lesion (uterus/ovary/others), blood transfusion (yes/no), neoadjuvant chemotherapy (NAC) (yes/no), smoking (yes/no), hypertension (present/absent), diabetes mellitus (present/absent), hyperlipidemia (present/absent), history of VTE (present/absent), varicose veins (present/absent), menopause (yes/no), hormone therapy (yes/no), prolonged immobility (yes/no), and history of malignant disease (present/absent). For measurement of lesion dimensions, endometrial cancer was measured as the uterine body, while the actual dimensions of cervical cancer, ovarian tumors, and solitary uterine myoma were measured. In patients with multiple uterine myomata, the largest transvers diameter of the entire uterus was measured.

Risk factors that were found to be significant by univariate analysis were subsequently assessed by multiple logistic regression analysis with forward selection of variables to identify significant independent determinants of the risk of VTE. Then a screening method for high-risk patients was devised by combining factors that were found to be independently related to the risk of VTE, and its sensitivity and specificity were evaluated by performing receiver operating characteristic (ROC) analysis.

All statistical analyses were carried out using SPSS software (PASWV19.0J).

## Results

Of the 183 patients enrolled, 23 patients (12.6%) were found to have VTE by preoperative and/or postoperative MDCT. Eighteen patients (9.8%) had preoperative VTE. DVT was located distal to the popliteal fossa (e.g., in the popliteal, tibial, fibular, or soleal veins) in 7 patients, proximal to the popliteal fossa (e.g., in the femoral or iliac veins, inferior vena cava, or pulmonary artery) in 5 patients, and affected both regions in 6 patients. Five other patients (2.7%) were found to have VTE on postoperative MDCT. None of these 5 patients had any evidence of VTE on preoperatively, but it was found when MDCT was performed prior to walking on the first day after the operation.

Of the 18 patients with preoperative VTE, 13 patients (72.2%) underwent surgery for malignancy, including ovarian cancer in 10 patients (55.6%), endometrial cancer in 2 patients (11.1%), and cervical cancer in 1 patient (5.6%). The other five patients (27.8%) underwent surgery for benign diseases, including uterine myoma in 4 patients (22.2%) and ovarian cyst (5.6%) in 1 patient. Among the 5 patients with postoperative VTE, 3 patients (60%) received surgery for malignancy (all 3 had ovarian cancer) and 2 patients (40%) had surgery for benign disease (both had uterine myoma).

Follow up of the 183 patients was performed between October 2002 and March 2012. Among the18 patients with preoperative VTE, there was one PE-related death (5.6%) while the patient was receiving chemotherapy. Seven patients (38.9%) died of cancer, one of whom developed VTE at three years and ten months postoperatively. The survivors were 5 patients (27.8%) with cancer and 5 patients (27.8%) with benign disease. The five patients who developed postoperative VTE all survived irrespective of whether they had cancer or benign disease. Among all 183 patients, there was one PE-related death (0.55%), while 30 patients (16.4%) died of cancer, one patient (0.55%) died of unknown causes, 51 patients (27.9%) survived with cancer, and 100 patients (54.6%) survived with benign diseases.

Univariate analysis (Mann-Whitney U test) revealed the following significant independent risk factors for VTE: PIC, TAT, and prolonged immobility (all p<0.001); age, malignancy, NAC, hypertension, history of VTE, and hormone therapy (all p<0.01); and hemoglobin, transverse tumor diameter, ovarian disease, and menopause (all p<0.05) (Table 3).

**Table 3:** Risk factors for venous thromboembolism.

Factor	p value
Age	0.006
Body mass index	n.s.
Thrombin-antithrombin III complex	0.000
Plasmin-alpha2-plasmin inhibitor complex	0.000
Hemoglobin	0.042
Hematocrit	n.s.
Platelet count	n.s.
Mean platelet volume	n.s.
International normalized ratio	n.s.
Longitudinal tumor diameter	n.s.
Anteroposterior tumor diameter	n.s.
Transverse tumor diameter	0.021
Lesion (uterus/ovary/others)	0.041
Malignancy	0.005
Operating time	n.s.
Blood loss	n.s.
Transfusion	n.s.
Neoadjuvant chemotherapy	0.004
Smoking	n.s.
Hypertension	0.003
Diabetes	n.s.
Hyperlipidemia	n.s.
History of venous thromboembolism	0.008
Varicose veins	n.s.
Menopause	0.010
Hormone replacement therapy	0.008
Prolonged immobility	0.000
History of malignancy	n.s.

Three factors were judged to be inappropriate for multivariate analysis because of the small sample size (history of VTE, hormone therapy, and prolonged immobility) and one factor was deleted due to unclear criteria (hypertension). Accordingly, multiple logistic regression analysis was performed by the forward selection method using PIC, TAT, hemoglobin, age, transverse tumor diameter, ovarian disease, malignancy, NAC, and menopause as independent variables, while VTE (yes/no) was the dependent variable. This analysis identified PIC (p<0.001), transverse tumor diameter (p<0.01), and age (p<0.05) as significant independent risk factors for VTE (Table 4).

**Table 4:** Results of multiple logistic regression analysis.

Factor	SEM	Probability	Odds Ratio	95% Confidence Interval	
				Lower limit	Upper limit
PIC	0.240	0.000	2.485	1.552	3.977
age	0.020	0.011	1.053	1.012	1.096
Transverse	0.044	0.005	1.130	1.037	1.232

Abbreviation: PIC, Plasmin-alpha2-plasmin inhibitor complex. Transverse, Transverse tumor diameter. SEM, Standard error of the mean (n = 183).

We then calculated the incidence rate of perioperative VTE in our gynecological patients using PIC and transverse tumor diameter stratified by age (Table 5). To devise a convenient algorithm for bedside assessment of VTE risk, we categorized the patients into 4 age groups, which were ≦39 years old, 40–49 years old, 50–59 years old, and ≧60 years old. We also divided PIC into 2 categories (≦1.2 µg/ml and ≧1.3 µg/ml) and transverse tumor diameter into 3 categories (≦9 cm, 10–14 cm, and ≧15 cm).

**Table 5:** Incidence rate of perioperative venous thromboembolism in patients groups stratified by age.

≦39 years old			
		PIC:≦1.2 µg/mL	PIC :≧1.3 µg/mL
Transverse:≦9 cm		1.5%	12.3%
Transverse:10–14 cm		2.6%	19.7%
Transverse:≧15 cm		8.2%	41.8%
Accuracy	93.6%		
40–49 years old			
		PIC:≦1.2 µg/mL	PIC :≧1.3 µg/mL
Transverse:≦9 cm		1.7%	14.0%
Transverse:10–14 cm		3.0%	22.2%
Transverse:≧15 cm		8.2%	45.4%
Accuracy	93.6%		
50–59 years old			
		PIC:≦1.2 µg/mL	PIC :≧1.3 µg/mL
Transverse:≦9 cm		2.7%	20.3%
Transverse:10–14 cm		4.6%	30.9%
Transverse:≧15 cm		12.3%	56.6%
Accuracy	78.2%		
≧60 years old			
		PIC:≦1.2 µg/mL	PIC :≧1.3 µg/mL
Transverse:≦9 cm		4.6%	30.9%
Transverse:10–14 cm		7.8%	44.0%
Transverse:≧15 cm		19.7%	69.7%
Accuracy	82.4%		

Abbreviation: PIC, Plasmin-alpha2-plasmin inhibitor complex. Transverse, Transverse tumor diameter.

Because a standard PIC value for diagnosis of VTE has not been established, we used ROC analysis to determine the optimal cut-off value. ROC analysis of the data showed that the optimum sensitivity and specificity were obtained at a PIC value of 1.25 µg/mL, with a sensitivity of 78.3%, specificity of 78.7%, positive predictive value of 78.3%, and area under the ROC curve (ROC-AUC) of 0.816. ROC analysis of the transverse tumor diameter revealed the optimum sensitivity and specificity at a diameter of 10.5 cm, with a sensitivity of 69.6%, specificity of 61.2%, and positive predictive value of 69.6%.

For patients ≦39 years old or aged 40–49 years who had a PIC level ≧1.3 µg/ml and a transverse tumor diameter ≧10 cm, the predicted incidence of perioperative VTE exceeded 20%, and the accuracy of this new screening method was 93.6%. For patients aged 50–59 years with a PIC ≧1.3 µg/ml, the predicted incidence of perioperative VTE was also >20% and the accuracy was 78.2%. For patients ≧60 years old with a transverse tumor diameter ≧15 cm (irrespective of PIC) or those with a PIC ≧1.3 µg/ml, the predicted incidence of perioperative VTE was >20% as well and the accuracy was 82.4%.

## Discussion

We devised a new screening method based on PIC, transverse tumor diameter, and age for predicting the risk of perioperative VTE in patients undergoing elective gynecological surgery.

PIC is a marker of the activation of fibrinolysis [3]. Its half-life is about 6 hours and it is a satisfactory indicator of an enhanced fibrinolytic state in vivo, particularly because it is almost undetectable in normal persons. Fibrinolytic activity refers to the so-called secondary fibrinolysis that occurs after a fibrin clot forms in vivo, so elevation of PIC provides evidence of thrombosis [4].

D-dimer is a degradation product of cross-linked fibrin from blood clots and is commonly employed as a highly sensitive (>95%) test for acute VTE that is very accurate for excluding this condition (its negative predictive value is 97%) [5], [6]. Measurement of D-dimer is particularly useful to exclude VTE in outpatients [2]. However, this test has a low specificity (30%) and low positive predictive value, which means that patients with high D-dimer levels do not necessarily have VTE [7]–[9], and there have been reports about no appreciable elevation of D-dimer during the stage of distal thrombus formation in patients with extensive thromboembolism [10]–[12]. Thus, measurement of D-dimer should only be used for excluding VTE.

Measurement of PIC enables assessment of fibrinolytic activity, and thus is expected to be more useful for the clinical evaluation of perioperative VTE, especially in patients who have a high risk of fatal postoperative PE and no evidence of preoperative VTE. The disadvantage of PIC is that one to two days is required to obtain the result, so this test is not suitable for emergency assessment.

The risk of VTE is strongly associated with age, and its incidence rises sharply with aging. Patients ≦39 years old generally have a low risk of VTE, while patients ≧40 old have a higher risk and the risk increases dramatically in patients ≧60 years old [13], [14]. Compared with the general population, the incidence of VTE is five-fold higher in patients aged ≧75 years (10 per 1000). With aging, decreased myodynamia and muscle tone in the lower limbs can lead to failure of venous pump function, resulting in a high rate of VTE in elderly patients [15], [16].

Unlike younger patients, elderly patients often do not have typical symptoms and imaging findings of VTE, suggesting that perioperative management needs to be modified according to age [17], [18].The majority of preventable deaths for VTE are ascribed to missed or late diagnosis rather than to failure of therapy [19].

Because an intrapelvic mass compresses the pelvic veins, a large ovarian tumor or uterine myoma can cause stasis of blood in the lower extremities, leading to the development of DVT in the venous sinuses of the soleus muscle or in the tibial and peroneal veins. Subsequent damage to the venous valves results in cephalad progression of the thrombus and the formation of free thrombi in the popliteal and femoral vein, which can be followed by the occurrence of PE.

Compared with the longitudinal and anteroposterior directions, compression of the pelvic veins by a mass lesion is more likely to occur in the transverse direction because of the relation to the pelvic bones. Vascular compression increases as a pelvic mass (especially an ovarian tumor) becomes larger, resulting in a high frequency of VTE. The size of a pelvic mass can be easily determined by imaging modalities clinically available to gynecologists.

The mortality of PE in untreated patients is reported to be as high as 30%, but this can be reduced to 2–8% by starting appropriate treatment, such as anticoagulant therapy, at an early stage [20].

Our follow up investigation of 23 patients with perioperative VTE revealed PE-related death during chemotherapy in only one patient, while the other 22 patients died of cancer or survived with malignant or benign diseases, so the cause of death was the primary disease and not VTE. This result emphasizes the importance of accurately diagnosing perioperative VTE at an early stage. If perioperative VTE is accurately diagnosed and treated early, it will generally be possible to prevent fatal PE. To achieve this objective, a convenient method of testing for perioperative VTE and assessing the risk factors for individual patients is essential because a delayed or missed diagnosis can result in death or long-term complications.

In the present study, we calculated the predicted incidence rate of perioperative VTE using the PIC level and the transverse tumor diameter stratified by age. Based on our findings, we propose the following algorithm for preoperative assessment of the risk of VTE.

After measurement of PIC and the transverse tumor diameter for screening, the incidence of VTE is predicted with adjustment for age. When the predicted risk of VTE is 20% or higher, MDCT should be performed within two weeks before surgery and prior to walking on the first postoperative day to detect proximal DVT and/or PE.

The main limitation of our study is that it was not designed to predict the actual incidence rate of perioperative VTE. Our screening method only extracts a high-risk group for perioperative VTE among patients having elective gynecological surgery. In order to actually identify VTE, it is necessary to perform further examinations such as MDCT in the high-risk group. Another limitation of our approach includes lack of data on important clinical symptoms for making a diagnosis of VTE such as pain, swelling, tenderness, and redness of the legs

In conclusion, PIC, transverse tumor diameter, and age are useful factors for predicting the risk of perioperative VTE. The new screening method that we developed is based on these three factors and can identify patients with a high risk of perioperative VTE/fatal PE. It is an easy method for gynecologists to adopt in daily clinical practice.
